# Creating an Age-Friendly Community in Affordable Housing Using a Resident Health Risk Assessment Focused on the 4Ms

**DOI:** 10.1093/geroni/igaa057.159

**Published:** 2020-12-16

**Authors:** Jennifer DeGennaro, Sherry Pomerantz, Margaret Avallone, Melonie Handberry, Elyse Perweiler

**Affiliations:** 1 RowanSOM, Stratford, New Jersey, United States; 2 Rutgers University-Camden, Camden, New Jersey, United States; 3 Fair Share Housing, Camden, New Jersey, United States; 4 Rowan University School of Osteopathic Medicine, Stratford, New Jersey, United States

## Abstract

The NJGWEP team in partnership with Fair Share Housing/Northgate II (NGII), an affordable housing complex in Camden, NJ, employed an iterative quality improvement process to collaboratively develop a Resident Health Risk Assessment (RHRA) to meet the needs of the housing facility and incorporate the essential elements of the 4Ms framework (Mentation, Medication, Mobility, and What Matters). Using the RHRA, NG II social services staff and Rutgers School of Nursing (RSoN) students were trained to collect health information and administer several evidence-based screening tools (i.e., MiniCog, TUG, PHQ-2). A final element of the RHRA still in development is the documentation process of referral and follow-up based on personalized care plans. Since July 2019, 43 RHRAs have been completed (60% female, mean age 66, age range=43 to 88). Almost all residents (94%) have at least 1 chronic condition (HTN, DM, COPD, CHF), although only 26% have an advance care plan. Most (81%) were screened for future fall risk; function (ADLs/IADLs) was assessed for all (100%). Every resident who was able or did not refuse (88%) was screened for cognitive impairment. Just 7% were taking a high-risk medication (i.e., an opioid or benzodiazepine). The NJGWEP team has initiated an age-friendly community at NGII by providing education on geriatric-focused topics and implementing the 4Ms-focused RHRA to detect issues impacting the resident’s well-being. Establishing a follow-up process to track referrals to available resources will enable NGII to allow residents to age in place with appropriate supports.

